# Identifying psychiatric morbidity and comorbidity patterns associated with COVID-19 mortality. A register-based cohort study from Catalonia

**DOI:** 10.1192/j.eurpsy.2025.10121

**Published:** 2025-11-19

**Authors:** Mireia Felez-Nobrega, Iago Giné-Vázquez, Anna Monistrol-Mula, Maria Melchior, Irwin Hecker, Ellenor Mittendorfer-Rutz, Katalin Gémes, Antonio Lora, Giulia Caggiu, Matteo Monzio, Claudia Conflitti, Marit Sijbrandij, Anke Witteveen, Josep Maria Haro

**Affiliations:** 1 Group of Epidemiology of Mental Disorders and Ageing, Sant Joan de Déu Research Institute, Esplugues de Llobregat, Barcelona, Spain; 2 Centro de Investigación Biomédica en Red de Salud Mental (CIBERSAM), Instituto de Salud Carlos III, Madrid, Spain; 3 Department of Medicine, Universitat de Barcelona, Barcelona, Spain; 4 Sorbonne Université, INSERM, Institut Pierre Louis d’Epidémiologie et de Santé Publique (IPLESP), Equipe de Recherche en Epidémiologie Sociale (ERES), Paris, France; 5 Division of Insurance Medicine, Department of Clinical Neuroscience, Karolinska Institutet, Stockholm, Sweden; 6 Department of Mental Health and Addition services, Local Health Authority (ASST) of Lecco, Lecco, Italy; 7 Department of Statistics and Quantitative Methods, University of Milano-Bicocca, Milan, Italy; 8 Department of Clinical, Neuro- and Developmental Psychology, WHO Collaborating Center for Research and Dissemination of Psychological Interventions, Amsterdam Public Health Research Institute, Vrije Universiteit Amsterdam, Amsterdam, The Netherlands

**Keywords:** clusters, COVID-19 mortality, mental disorders, psychiatric morbidity

## Abstract

**Background:**

There is limited evidence on the association between psychiatric morbidity and COVID-19 mortality.

**Methods:**

We used deidentified electronic health records from the Catalan public health system to evaluate the association between number of mental disorders and COVID-19 mortality. Adults diagnosed with a mental disorder in Catalonia’s mental health services (from to 2017–2019) were compared to a matched (1:1) control group by sex, age, and area of residence. COVID-19 mortality risk was evaluated from February to December 2020. Odds ratios (OR) with 95% confidence intervals (CI) were estimated for the association between the number of mental disorders and COVID-19 mortality. To examine if different patterns of psychiatric comorbidity were related to COVID-19 death, we performed K-means cluster analysis on individuals with ≥2 disorders, stratified by COVID-19 death.

**Results:**

The final sample included 785,378 adults (392,689 with ≥1 mental disorder). Mortality risk increased with the number of mental disorders: OR 1.23 (95% CI: 1.11–1.35) for one mental disorder, up to 5.21 (95% CI: 1.34–20.27) for four or more. Cluster analysis (n=84,207) identified seven psychiatric comorbidity profiles among those who did not die of COVID-19, and six profiles among those who died, with substantial comparability between cohorts.

**Conclusion:**

An increasing number of psychiatric diagnoses was associated with greater COVID-19 mortality, while specific comorbidity patterns showed limited differential influence. This suggests that it is not the specific combination of mental disorders that influences COVID-19 death outcomes, but rather the overall burden of multiple diagnoses.

## Introduction

Psychiatric comorbidity refers to the simultaneous presence of one or more additional mental disorders alongside a primary mental disorder [1]. This phenomenon is very common, and convincing evidence indicates that individuals diagnosed with a mental disorder are at a higher risk of developing additional mental health conditions in the future [2, 3]. Psychiatric comorbidity significantly affects overall health, often leading to complex clinical presentations, challenging treatment scenarios, and worse outcomes than those with a single diagnosis [4–7]. For instance, individuals with comorbid depression and anxiety tend to experience more severe symptoms, a longer course of illness, greater functional disability, and a lower response to treatment compared to those with only depression or anxiety [8–11]. Moreover, the presence of multiple mental disorders usually correlates with poorer physical outcomes [12]. In fact, individuals with two or more mental disorders have a shorter life expectancy than those with only one [13].

While psychiatric comorbidity is a significant risk factor for later health outcomes, literature on how it may influence COVID-19 mortality remains scarce, since most past evidence has restricted the focus on the presence of a single mental condition. In this context, evidence from the latest meta-analysis suggests that, compared to individuals without a mental disorder, those with a preexisting mental disorder have a disproportionately higher risk of COVID-19 death [14]. However, to the best of our knowledge, no studies have yet addressed comorbidity across mental disorders and COVID-19 outcomes, except for one brief report [15]. This study based on the United States, compared the odds for several COVID-19 outcomes, between those with internalizing, externalizing, and thought disorders and their co-occurrence. The authors found that the co-occurrence of any two mental diagnostic groups was associated with an increased risk of COVID-19 hospitalization and death [15]. While this evidence is informative, a more robust framework for addressing how psychiatric comorbidity influences COVID-19 outcomes is needed. Accounting for psychiatric comorbidity in COVID-19 research has been recommended since the early phases of the pandemic [16], and this information is essential for advancing our understanding of patients’ risk, and for improving patient outcomes through better preventive strategies to best prepare for future sanitary crises. Therefore, our study aimed to analyze the impact of psychiatric comorbidity on COVID-19 death, and to explore profiles through clustering of mental disorders in those who died and did not die due to COVID-19.

## Methods

This register-based cohort study was conducted using deidentified electronic health records from Catalonia, Spain. Data were retrieved from the Health Quality and Assessment Agency of Catalonia, which is responsible for managing the Public Data Analysis for Health Research and Innovation Programme [17]. Health registers used the ninth and tenth versions of the International Classification of Diseases (ICD-9/ICD-10).

In adherence to the present regulations governing the utilization of registry-based health data, obtaining informed consent was deemed unnecessary. The ethics committee of Fundació Sant Joan de Déu (PIC-160-21) granted approval for this study.

### Preexisting mental disorders

We selected all adults (≥18 years in 2017) who were alive on December 31, 2019, and who received specialized inpatient or outpatient mental health care between January 1, 2017 and December 31, 2019, for one of the following mental disorders: non-affective psychosis, bipolar disorder, depressive disorder, stress-related disorders, neurotic/somatoform disorders, and substance misuse (ICD-9 and ICD-10 codes in Supplementary Materials). Every person diagnosed with one of the specified mental disorders (exposed) was matched (1:1) to a randomly selected individual from the Catalonia health registry on sex, age within 3-year bands, and residential area, who had no specialized inpatient or outpatient mental health diagnosis between January 1, 2017 and December 31, 2019 (unexposed).

In order to account for psychiatric comorbidities, the groups of mental disorders are not mutually exclusive, and individuals may be categorized into multiple groups if they have more than one mental diagnosis. For some analyses, we also created a five-category variable to represent the number of preexisting mental disorders (0, 1, 2, 3, ≥4). This variable was formed by aggregating the mental disorders groups previously identified.

### Outcome measures and control variables

COVID-19-related death (outcome) was ascertained using mortuary records, and a dichotomous variable was created (yes/no). In all analyses, we accounted for sex (women vs. men), age (18–30 years, 31–40, 41–50, 51–60, 61–70, 71–80, ≥81), physical diagnoses, and nursing home residency. Data on physical diseases were obtained from primary care registries between 1997 and 2018 (the most recent data that were available at the time of data extraction). Physical diseases included those that have been related to severe COVID-19 outcomes including asthma, cardiovascular diseases, chronic pulmonary disease, diabetes, dyslipidemia, heart failure, hypertension, ischemic heart disease, malignant neoplasia, and obesity (ICD-10 codes in Supplementary Materials). A three-level variable was created: 0, 1, ≥2. Nursing home residency (yes vs. no) included those who stayed in nursing homes at some point between February 25, 2020 (the date of the first official reported case of COVID-19 in Catalonia) and December 31, 2020 (end point of study period).

### Statistical analysis

Differences in sample characteristics by COVID-19-related death were tested by Chi-squared test. Using the overall sample, conditional logistic regression analysis was conducted to assess the association between the number of mental disorders and COVID-19-related death. Model 1 included only the number of mental disorders, whereas Model 2 additionally adjusted for the number of physical diseases and nursing home residency. Results from the logistic regression analyses are presented as odds ratios (ORs) with 95% confidence intervals (CIs). Next, to assess psychiatric comorbidity patterns associated with COVID-19 mortality, we conducted a k-means cluster analysis [18] restricting to those who were diagnosed with ≥2 mental disorders and among those with and without COVID-19 death separately. As a preliminary step, continuous coordinates for individuals were drawn from the discrete mental conditions by means of multiple correspondence analysis (MCA). Subsequently, we applied k-means cluster analysis to these new dimensions in order to detect patterns of mental conditions through the clusters of individuals. We determined the optimum number of clusters based on the Calinski–Harabasz index [19], the Silhouette index (computed both as average of cluster silhouettes and individual silhouettes) [20], the within-cluster sum of squares, and the logarithm of the sum of squares ratio [21, 22] (for a more detailed description, see the Supplementary Materials). We characterized clusters based on variables with observed/expected ratio (i.e., the ratio of the proportion in each cluster against the proportion in the whole group) exceeding 1.2 or falling behind 0.8. Additionally, clusters were defined by mental disorders where over 70% of patients exhibiting that condition were present within the cluster, or over 70% of patients in the cluster exhibited the disorder. More information regarding the statistical procedures can be found in the Supplementary Materials. The statistical analysis was done with R version 4.4.0 [23] on Windows 10×64 (build 19045) and the package FactoMineR [24] was used for the MCA.

## Results

The final sample consisted of 785,378 adults aged ≥18 years. Overall, 392,689 people were diagnosed with a mental disorder of interest between January 1, 2017 and December 31, 2020. A total of 308,482 (39.3%) were identified as having one mental disorder of interest while 3,114 (0.4%) participants were classified as having ≥4 mental diagnoses. Participants who were more likely to die from COVID-19 were those with ≥2 physical diseases and those who stayed in a nursing home. In the sample restricted to individuals with two or more mental disorders, the most common mental diagnoses were neurotic and somatoform disorders (n = 49,881; 59.2%), substance misuse (n = 48,077; 57.1%), and depressive disorder (n = 42,982; 51%). More information on the sample characteristics is provided in Table 1. Compared with participants without mental conditions, the risk of mortality due to COVID-19 increased with each additional comorbid mental disorder present (Table 2). Adjusting for the number of physical diseases and nursing home residency slightly attenuated the OR, except for individuals with four or more mental disorders, for whom the risk estimate increased further (Table 2, Model 2).

**Table 1.** Sample characteristics (overall and by COVID-19-related death)

%		Overall (n = 785,378)	COVID death NO (n = 782,794)	COVID death YES (n = 2,584)	p-value
Sex	Men	42.9	42.9	46.8	<0.001
	Women	57.1	57.1	53.2	
Age	18–30	9.4	9.4	0.1	<0.001
	31–40	12.2	12.2	0.3	
	41–50	19.2	19.2	2.0	
	51–60	21.2	21.3	6.2	
	61–70	18.8	18.8	15.5	
	71–80	13.2	13.1	31.9	
	≥81	6.1	6.0	44.0	
No. mental disorders	0	50.0	50.0	41.1	<0.001
	1	39.3	39.3	45.1	
	2	8.3	8.3	11.0	
	3	2.0	2.0	2.3	
	≥4	0.4	0.4	0.5	
Non-affective psychosis^a^	No	81.0	81.0	78.2	0.194
	Yes	19.0	19.0	21.8	
Bipolar disorder^a^	No	89.5	89.5	85.7	0.026
	Yes	10.5	10.5	14.3	
Depressive disorder^a^	No	49.0	49.0	37.3	<0.001
	Yes	51.0	51.0	62.7	
Stress-related disorder^a^	No	70.6	70.5	87.4	<0.001
	Yes	29.4	29.5	12.6	
Neurotic and somatoform disorders^a^	No	40.8	40.8	43.1	0.389
	Yes	59.2	59.2	56.9	
Substance misuse^a^	No	42.9	42.9	44.5	0.568
	Yes	57.1	57.1	55.5	
No. physical diseases	0	44.3	44.4	16.2	<0.001
	1	23.5	23.5	15.6	
	≥2	32.2	32.1	68.3	
Nursing home residency	No	97.9	98.0	72.4	<0.001
	Yes	2.1	2.0	27.6	

a Data are shown for the sample restricted to those with ≥2 mental disorders (n = 84,207). The groups are not mutually exclusive, as individuals may be categorized into multiple groups if they have more than one mental diagnosis.

**Table 2.** Association between number of mental disorders and COVID-19-related mortality estimated by multivariable conditional logistic regression

Characteristic		Model 1		Model 2	
		OR	[95% CI]	OR	[95% CI]
No. mental disorders	0	1.00		1.00	
	1	1.29	(1.18, 1.41)	1.23	(1.11, 1.35)
	2	2.26	(1.83, 2.78)	1.87	(1.49, 2.35)
	3	2.52	(1.56, 4.09)	2.13	(1.28, 3.53)
	≥4	4.00	(1.13, 14.17)	5.21	(1.34, 20.27)
No. physical diseases	0			1.00	
	1			1.15	(0.92, 1.45)
	≥2			1.54	(1.26, 1.87)
Nursing home residency	Yes vs. No			7.42	(5.83, 9.46)

Abbreviations: CI, confidence interval; OR, odds ratio.

**Figure 1.**
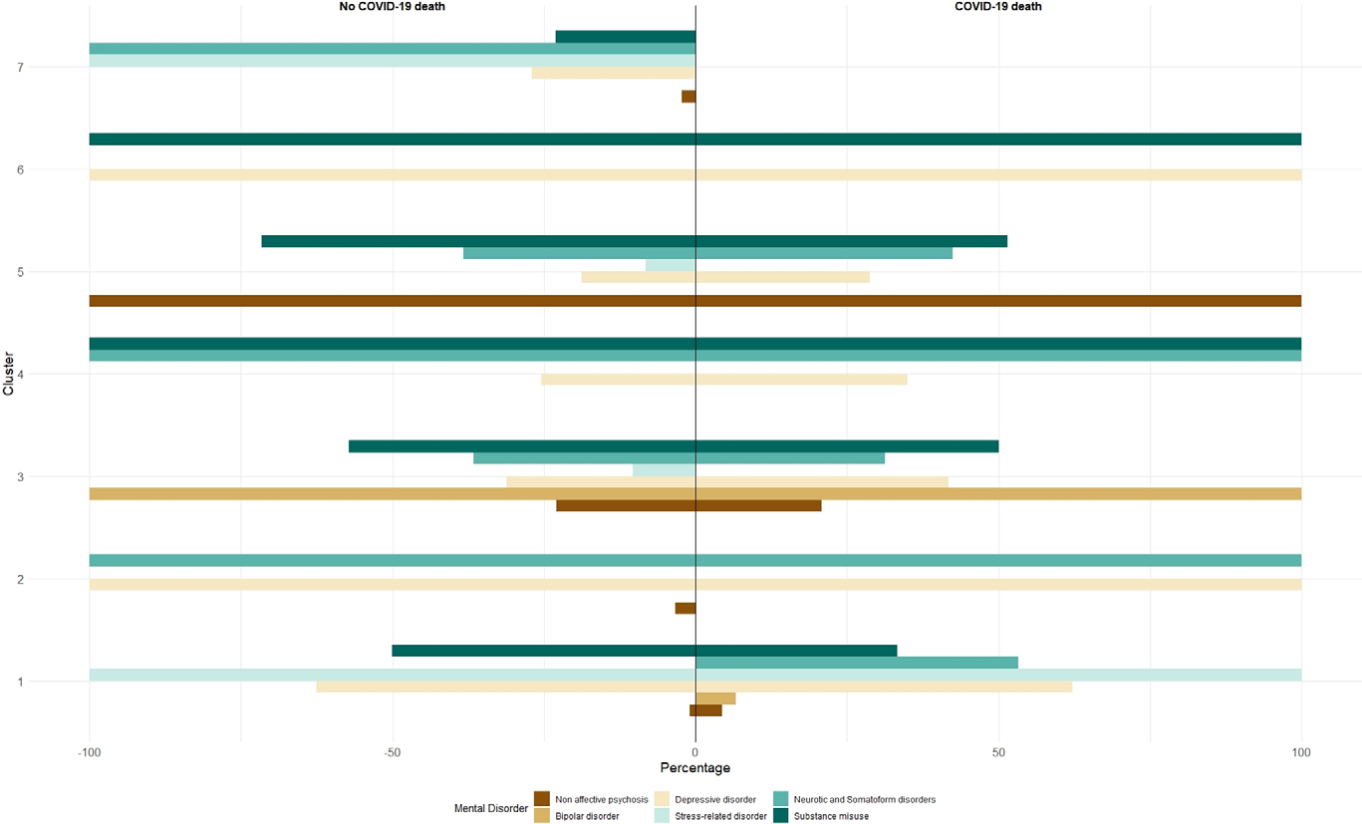
Percentage of patients in each cluster diagnosed with the mental disorders.

In the cluster analysis conducted in the subsample of ≥2 mental disorders (n = 84,207), we identified seven profiles of psychiatric comorbidities among those who did not die from COVID-19 (Table 3, Figure 1) and six profiles among those who died from COVID-19 (Table 4, Figure 1). All clusters identified were small, and patient groups exhibited a high degree of homogeneity in terms of distributions across clusters (10.52–18.90% for those who did not die and 12.61–20.45% for those who died from COVID-19).

**Table 3.** Profiles of mental disorders identified in k-means cluster analysis

COVID-related death: NO (N = 83,850)		
Cluster description	Observed in cluster/expected >1.2 or <0.8	High-frequency variables, n (%)
**Cluster 1**		
N (%) 11,146 (13.29) Women, N (%) 7,008 (62.9) Age, N (%) 18–30 = 1136 (10.2) 31–40 = 1195 (10.7) 41–50 = 2463 (22.1) 51–60 = 2947 (26.4) 61–70 = 2145 (19.2) 71–80 = 966 (8.7) ≥81 = 294 (2.6)	Mental disorders High: Depressive disorder (1.23), Stress-related disorders (3.39) Low: Non-affective psychosis (0.05), Bipolar disorder (0.0), Neurotic and somatoform disorders (0.0) Demographics and other factors High: None Low: Age ≥ 81 (0.73), Nursing home residents (0.56)	Cluster contains ≥70% of all patients with this variable: None ≥70% of patients in this cluster have this variable: Stress-related disorders (100%)
**Cluster 2**		
N (%) 14,024 (16.73) Women, N (%) 10838 (77.3) Age, N (%) 18–30 = 830 (5.9) 31–40 = 1055 (7.5) 41–50 = 2200 (15.7) 51–60 = 3007 (21.4) 61–70 = 3047 (21.7) 71–80 = 2506 (17.9) ≥81 = 1379 (9.8)	Mental disorders High: Depressive disorder (1.96), Neurotic and somatoform disorders (1.69) Low: Non-affective psychosis (0.18), Bipolar disorder (0.0), Stress-related disorders (0.0), Substance misuse (0.0). Demographics and other factors High: Female (1.32), Age 71–80 (1.76), Age ≥ 81 (2.7), 2+ Physical diseases (1.28) Low: Male (0.55), Age 18–30 (0.64), Age 31–40 (0.59), Age 41–50 (0.72), 0 Physical diseases (0.77)	Cluster contains ≥70% of all patients with this variable: None ≥70% of patients in this cluster have this variable: Depressive disorder (100%); Neurotic and somatoform disorders (100%)
**Cluster 3**		
N (%) 8,822 (10.52) Women, N (%) 4852 (55.0) Age, N (%) 18–30 = 741 (8.4) 31–40 = 1166 (13.2) 41–50 = 2000 (22.7) 51–60 = 2224 (25.2) 61–70 = 1642 (18.6) 71–80 = 848 (9.6) ≥81 = 201 (2.3)	Mental disorders High: Non-affective psychosis (1.21), Bipolar disorder (9.5) Low: Depressive disorder (0.61), Stress-related disorder (0.35), Neurotic and somatoform disorders (0.62) Demographics and other factors High: None Low: Age ≥ 81 (0.63)	Cluster contains ≥70% of all patients with this variable: Bipolar disorder (100%) ≥70% of patients in this cluster have this variable: Bipolar disorder (100%)
**Cluster 4**		
N (%) 15,846 (18.90) Women, N (%) 8224 (51.9) Age, N (%) 18–30 = 1285 (8.1) 31–40 = 2328 (14.7) 41–50 = 3706 (23.4) 51–60 = 4066 (25.7) 61–70 = 2841 (17.9) 71–80 = 1349 (8.5) ≥81 = 271 (1.7)	Mental disorders High: Neurotic and somatoform disorders (1.69), Substance misuse (1.75) Low: Non-affective psychosis (0.0); Bipolar disorder (0.0), Depressive disorder (0.5), Stress-related disorder (0.0) Demographics and other factors High: None Low: Age ≥ 81 (0.47), Nursing home residents (0.74)	Cluster contains ≥70% of all patients with this variable: None ≥70% of patients in this cluster have this variable: Neurotic and somatoform disorders (100%); Substance misuse (100%)
**Cluster 5**		
N (%) 13,062 (15.58) Women, N (%) 4828 (37.0) Age, N (%) 18–30 = 1839 (14.1) 31–40 = 2479 (19.0) 41–50 = 3294 (25.2) 51–60 = 2693 (20.6) 61–70 = 1612 (12.3) 71–80 = 824 (6.3) ≥81 = 321 (2.5)	Mental disorders High: Non-affective psychosis (5.26), Substance misuse (1.25) Low: Bipolar disorder (0.0), Depressive disorder (0.37), Stress-related disorder (0.28), Neurotic and somatoform disorder (0.65) Demographics and other factors High: Male (1.51), Age 18–30 (1.53), Age 31–40 (1.5), 0 Physical diseases (1.25), Nursing home residents (1.99) Low: Female (0.63), Age 61–70 (0.67), Age 71–80 (0.62), Age ≥ 81 (0.68), 2+ Physical diseases (0.77)	Cluster contains ≥70% of all patients with this variable: Non-affective psychosis (81.98%) ≥70% of patients in this cluster have this variable: Non-affective psychosis (100%); Substance misuse (71.6%)
**Cluster 6**		
N (%) 9,370 (11.17) Women, N (%) 5246 (56.0) Age, N (%) 18–30 = 386 (4.1) 31–40 = 681 (7.3) 41–50 = 1506 (16.1) 51–60 = 2714 (29.0) 61–70 = 2575 (27.5) 71–80 = 1234 (13.2) ≥81 = 274 (2.9)	Mental disorders High: Depressive disorder (1.96), Substance misuse (1.75) Low: Non-affective psychosis (0.0), Bipolar disorder (0.0), Stress-related disorders (0.0), Neurotic and somatoform disorders (0.0) Demographics and other factors High: Age 61–70 (1.5), Age 71–80 (1.3), 2+ Physical diseases (1.22) Low: Age 18–30 (0.45), Age 31–40 (0.57), Age 41–50 (0.74), Nursing home residents (0.78)	Cluster contains ≥70% of all patients with this variable: None ≥70% of patients in this cluster have this variable: Depressive disorder (100%); Substance misuse (100%)
**Cluster 7**		
N (%) 11,580 (13.81) Women, N (%) 7947 (68.6) Age, N (%) 18–30 = 1516 (13.1) 31–40 = 1717 (14.8) 41–50 = 2993 (25.8) 51–60 = 2748 (23.7) 61–70 = 1515 (13.1) 71–80 = 782 (6.8) ≥81 = 309 (2.7)	Mental disorders High: Stress-related disorders (3.39), Neurotic and somatoform disorders (1.69) Low: Non-affective psychosis (0.12), Bipolar disorder (0.0), Depressive disorder (0.53), Substance misuse (0.4) Demographics and other factors High: Age 18–30 (1.42) Low: Male (0.75), Age 61–70 (0.71), Age 71–80 (0.67), Age ≥ 81 (0.73), Nursing home residents (0.7)	Cluster contains ≥70% of all patients with this variable: None ≥70% of patients in this cluster have this variable: Stress-related disorders (100%); Neurotic and somatoform disorders (100%)

*Note:* The analyses were conducted in the sample restricted to those with ≥2 mental disorders.

**Table 4.** Profiles of mental disorders identified in k-means cluster analysis

COVID-related death: YES (N = 357)		
Cluster description	Observed in cluster/expected >1.2 or <0.8	High-frequency variables, n (%)
**Cluster 1**		
N (%) 45 (12.61) Women, N (%) 27 (60.0) Age, N (%) 18–30 = 0 31–40 = 1 (2.2) 41–50 = 3 (6.7) 51–60 = 6 (13.3) 61–70 = 7 (15.6) 71–80 = 14 (31.1) ≥81 = 14 (31.1)	Mental disorders High: Stress-related disorder (7.93), Low: Non-affective psychosis (0.2), Bipolar disorder (0.47), Substance misuse (0.6) Demographics and other factors High: Age 31–40 (2.64), Age 41–50 (1.7), Age 51–60 (1.32) Low: Age 61–70 (0.62), Nursing home residents (0.54)	Cluster contains ≥70% of all patients with this variable: Stress-related disorders (100%) ≥70% of patients in this cluster have this variable: Stress-related disorders (100%)
**Cluster 2**		
N (%) 73 (20.45) Women, N (%) 59 (80.8) Age, N (%) 18–30 = 0 31–40 = 1 (1.4) 41–50 = 0 (0.0) 51–60 = 1 (1.4) 61–70 = 17 (23.3) 71–80 = 26 (35.6) ≥81 = 28 (38.4)	Mental disorders High: Depressive disorder (1.59), Neurotic and somatoform disorders (1.76), Low: Non-affective psychosis (0.0), Bipolar disorder (0.0), Stress-related disorders (0.0), Substance misuse (0.0) Demographics and other factors High: Female (1.55), Age 31–40 (1.63), Age ≥81 (1.46) Low: Male (0.4), Age 41–50 (0.0), Age 51–60 (0.14), 0 Physical diseases (0.38)	Cluster contains ≥70% of all patients with this variable: None ≥70% of patients in this cluster have this variable: Depressive disorder (100%); Neurotic and somatoform disorders (100%)
**Cluster 3**		
N (%) 48 (13.45) Women, N (%) 23 (47.9) Age, N (%) 18–30 = 0 31–40 = 0 41–50 = 3 (6.2) 51–60 = 6 (12.5) 61–70 = 13 (27.1) 71–80 = 18 (37.5) ≥81 = 8 (16.7)	Mental disorders High: Bipolar disorder (7), Low: Depressive disorder (0.66), Stress-related disorder (0.0), Neurotic and somatoform disorders (0.55) Demographics and other factors High: Age 41–50 (1.59), Age 51–60 (1.24), 0 Physical diseases (1.29), Nursing home residents (1.52) Low: Age 31–40 (0.0), Age ≥ 81 (0.63)	Cluster contains ≥70% of all patients with this variable: Bipolar disorder (94.12%) ≥70% of patients in this cluster have this variable: Bipolar disorder (100%)
**Cluster 4**		
N (%) 63 (17.65) Women, N (%) 23 (36.5) Age, N (%) 18–30 = 0 31–40 = 0 41–50 = 5 (7.9) 51–60 = 11 (17.5) 61–70 = 22 (34.9) 71–80 = 13 (20.6) ≥81 = 12 (19.0)	Mental disorders High: Neurotic and somatoform disorders (1.76), Substance misuse (1.8) Low: Non-affective psychosis (0.0), Bipolar disorder (0.0), Depressive disorder (0.56), Stress-related disorder (0.0) Demographics and other factors High: Male (1.33), Age 41–50 (2.02), Age 51–60 (1.73), Age 61–70 (1.39) Low: Female (0.7), Age 31–40 (0.0), Age 71–80 (0.61), Age ≥ 81 (0.72), Nursing home residents (0.58)	Cluster contains ≥70% of all patients with this variable: None ≥70% of patients in this cluster have this variable: Neurotic and somatoform disorders (100%); Substance misuse (100%)
**Cluster 5**		
N (%) 66 (18.49) Women, N (%) 29 (43.9) Age, N (%) 18–30 = 0 31–40 = 1 (1.5) 41–50 = 1 (1.5) 51–60 = 7 (10.6) 61–70 = 9 (13.6) 71–80 = 26 (39.4) ≥81 = 22 (33.3)	Mental disorders High: Non-affective psychosis (4.58), Low: Bipolar disorder (0), Depressive disorder (0.46), Stress-related disorders (0.0), Neurotic and somatoform disorders (0.75) Demographics and other factors High: Age 31–40 (1.8), Age ≥ 81 (1.27), 1 Physical diseases (1.5), Nursing home residents (1.66) Low: Age 41–50 (0.39), Age 61–70 (0.54), 0 Physical diseases (0.73), Non-Nursing home residents (0.78)	Cluster contains ≥70% of all patients with this variable: Non-affective psychosis (84.62%) ≥70% of patients in this cluster have this variable: Non-affective psychosis (100%)
**Cluster 6**		
N (%) 62 (17.37) Women, N (%) 25 (40.3) Age, N (%) 18–30 = 0 31–40 = 0 41–50 = 2 (3.2) 51–60 = 5 (8.1) 61–70 = 22 (35.5) 71–80 = 23 (37.1) ≥81 = 10 (16.1)	Mental disorders High: Depressive disorder (1.59), Substance misuse (1.8) Low: Non-affective psychosis (0.0), Bipolar disorder (0.0), Stress-related disorders (0.0), Neurotic and somatoform disorders (0.0) Demographics and other factors High: Male (1.25), Age 61–70 (1.41), 0 Physical diseases (1.55) Low: Female (0.77), Age 31–40 (0.0), Age 51–60 (0.8), Age ≥ 81 (0.61), Nursing home residents (0.72)	Cluster contains ≥70% of all patients with this variable: None ≥70% of patients in this cluster have this variable: Depressive disorder (100%); Substance misuse (100%)

*Note:* The analyses were conducted in the sample restricted to those with ≥2 mental disorders.

Clusters in both cohorts showed a significant degree of comparability. That is, the first cluster identified (Cluster 1) was common in those who did not die due to COVID-19 and those who died (13.29, 12.61%). This cluster was characterized by individuals with stress-related disorders, and around 62% of patients of this cluster also had depression (Figure 1). We identified a second cluster (Cluster 2) in both cohorts (16.73% of those who did not die from COVID-19, and 20.45% of the comparator cohort) characterized by patients with depressive disorder and neurotic/somatoform disorders and with high prevalence of older age (≥81 years) and females. We also found a cluster in both cohorts (Cluster 3, 10.52 and 13.45%) that consisted almost exclusively of individuals with bipolar disorder, and showed a low prevalence of older age (≥81 years). Cluster 4 was also present in both cohorts (18.90% vs. 17.65%) and was characterized by those who had substance misuse and neurotic/somatoform disorders with a low prevalence of older age and nursing home residents. Another cluster (Cluster 5, 15.85 and 18.49%) that was common to both cohorts was distinguished by patients having a diagnosis of non-affective psychosis, and an important collective of those had substance misuse (especially among those who did not die from COVID-19). In addition, there was a high prevalence of nursing home residents in this cluster. Finally, we also identified another cluster in both groups characterized by patients with depression and substance misuse (Cluster 6, 11.17 and 17.37%). This cluster was associated with advanced age (61–70 years) but with a low prevalence of nursing home residents. We also identified another cluster of individuals unique to those who did not die due to COVID-19, characterized by individuals with stress-related disorders and neurotic/somatoform disorders (Cluster 7, Table 4). Interestingly, this cluster can be comparable to some extent to the Cluster 1 identified in those who died due to COVID-19, as 53.33% of patients located in this cluster also had neurotic/somatoform disorders (Figure 1). However, although Cluster 1 (COVID-19 death cohort) also included a high prevalence of depression, this did not occur in Cluster 7. Additionally, both Cluster 1 (in both cohorts) and Cluster 7 were associated with a low prevalence of nursing home residents.

## Discussion

In the current study based on register-health data from Catalonia (Spain), we assessed the association of having multiple mental diagnoses with COVID-19 death, and we explored patterns of psychiatric comorbidity in individuals who died and did not die from COVID-19.

Our results showed that the number of mental health diagnoses was associated with a higher risk of COVID-19 death in a dose–response manner. These findings partly contrast with those of a study that examined the risk of COVID-19 death among individuals with various mental disorders. That study found that individuals with internalizing and thought disorders, as well as those with externalizing and thought disorders, faced a higher risk of COVID-19 mortality compared with those without a mental disorder. However, the authors did not observe an elevated risk for individuals with internalizing and externalizing disorders or for those with the highest level of psychiatric comorbidity (internalizing, externalizing, and thought disorders) [15]. Nevertheless, our results align with several other studies reporting worse health outcomes and excess mortality in individuals with multiple mental health diagnoses [7, 13].

Several mechanisms might underlie the relationship between a higher number of psychiatric comorbidities and increased COVID-19 mortality. Chronic stress, commonly associated with mental disorders, can lead to systemic physiological effects, such as increased inflammation and a weakened immune response [25], which contribute to COVID-19 severity and mortality [26]. It is also possible that each additional mental disorder compounds the overall psychological burden and stress, further impairing the immune system and thereby increasing the risk of death from COVID-19. Furthermore, people with psychiatric comorbidities might face significant challenges in navigating the health care system and often experience fragmented care [27]. These difficulties can lead to delays in seeking and receiving treatment, thereby increasing their risk of mortality. Additionally, managing multiple mental disorders typically requires more complex and comprehensive treatment strategies [5, 11, 28]. This complexity can challenge achieving optimal treatment adherence, especially when combined with physical health conditions [29]. Inadequate or inconsistent treatment can result in poorer health outcomes and a higher risk of COVID-19 death. Finally, having multiple mental disorders might also exacerbate social and economic challenges, such as unemployment, social isolation, and poverty [30]. These factors can limit access to health care and other essential resources for maintaining good health, thereby contributing to increased COVID-19 mortality. In summary, the observed dose–response relationship suggests that each additional mental health diagnosis increases the risk of COVID-19 death, highlighting the need for comprehensive health assessments and targeted interventions for individuals with psychiatric comorbidities.

We also investigated the clustering of psychiatric comorbidities in individuals who died and those who did not die of COVID-19. Several clusters (i.e., Clusters 1, 2, and 7) were characterized by comorbidities between depression, neurotic/somatoform disorders, and stress-related disorders, which is in line with the results from a previous registry-based study. This population study, tracking over 7.5 million individuals from 1995 to 2016, found that mood disorders and neurotic, stress-related, or somatoform disorders frequently co-occurred. Specifically, these conditions were present in 80% of individuals with two mental disorders, 92% of those with three disorders, and 98% of those with four or more disorders [31]. Additionally, our analysis revealed that substance use disorders had an important contribution to comorbidity. Various clusters (i.e., Clusters 4–6) were defined by a specific disorder (neurotic/somatoform disorders, non-affective psychosis, and depression, respectively) combined with a high prevalence of comorbid substance use disorders. Indeed, previous evidence suggests that the comorbidity between substance use disorders and mood or anxiety disorders is highly prevalent [32], and 42% of individuals with psychotic disorders have been reported to also present substance use disorder [33]. This is particularly significant, given that combinations of mental disorders involving substance use disorders are associated with the highest levels of excess mortality [31]. Tobacco use is of special concern, as tobacco-related diseases have been reported to be responsible for around 50% of deaths among individuals with mental disorders such as schizophrenia and bipolar disorder [34]. Therefore, interventions that address smoking cessation in this population are essential for reducing their excess mortality and improving overall health outcomes [35].

Interestingly, our findings indicated that the clustering of mental disorders was similar for those who died of COVID-19 and for those who did not. The most recent meta-analysis that explored COVID-19 mortality according to the type of mental disorder found that almost all the mental disorders that were included, (with the exception of anxiety and neurodevelopmental disorders), were associated with an increased risk of COVID-19 death [14]. Thus, while we found that an increasing number of mental disorders was associated with progressively higher odds of COVID-19 death, the specific patterns of mental health comorbidities might not influence COVID-19 death. However, further research is needed to corroborate present findings.

To the best of our knowledge, this study is the first to analyze the impact of psychiatric comorbidity on COVID-19 mortality and to explore the clustering of mental disorders in those who died and in those who did not die due to COVID-19. We present novel insights into the implications of psychiatric comorbidity, a subject of immense significance due to its widespread occurrence and its substantial impact on public health.

A key strength of our study lies in the utilization of a comprehensive dataset derived from electronic health records, which offers a solid basis for generating real-world evidence that accurately reflects everyday clinical practices. Nevertheless, this study should be considered in the light of several limitations. First, as with all studies using electronic health records, potential biases in recording variables might exist. Second, mental disorders were defined based on ICD-9/10 codes among patients receiving specialized inpatient and outpatient mental health care from 2017 to 2019. Consequently, our study lacks information on the current symptomatology or severity of these disorders. Third, to make the analyses manageable, we focused on broad diagnostic categories rather than individual diagnoses. As a result, our findings might have differed if we had considered alternative groupings of mental disorders. Nevertheless, our categorizations were consistent with the literature. Fourth, our data did not capture all mental health diagnoses, potentially underestimating the number of individuals with mental disorders and the prevalence of psychiatric comorbidities. Fifth, we were unable to control for body mass index, other clinical variables, tobacco smoking, and socioeconomic status, which might confound the associations between psychiatric comorbidities and COVID-19-related death [36–38]. Sixth, although we adjusted for the number of physical diseases, we did not include individual disorders as separate covariates. This was done to keep the analyses tractable and avoid overfitting, but it limits our ability to detect potentially heterogeneous effects of specific conditions. In addition, the data used in this study did not include psychiatric medication or COVID-19 treatment information, which might influence mortality risk. Finally, the sample size was limited for certain groups of psychiatric comorbidities and for the entire subgroup of COVID-19 deaths, which affected the clustering analysis.

In conclusion, we found that the risk of COVID-19 death increased with the number of psychiatric comorbidities, which highlights the critical need for comprehensive care models that address the complex interplay of multiple mental health conditions. However, we did not find significant differences in the clustering of psychiatric comorbidities in those who died and in those who did not die of COVID-19. This suggests that it is not the specific combination of mental disorders that influences COVID-19 death outcomes, but rather the overall burden of multiple diagnoses.

## Supporting information

10.1192/j.eurpsy.2025.10121.sm001 Felez-Nobrega et al. supplementary material Felez-Nobrega et al. supplementary material

## Data Availability

Data were obtained from a third party and are not publicly available.

## Supplementary material

The supplementary material for this article can be found at http://doi.org/10.1192/j.eurpsy.2025.10121.
