# Supplementary material for: Identifying psychiatric morbidity and comorbidity patterns associated with COVID-19 mortality. A register-based cohort study from Catalonia
Source: Eur Psychiatry. 2025 Nov 19;68(1):e170. doi: 10.1192/j.eurpsy.2025.10121 (PMC12721987; doi:10.1192/j.eurpsy.2025.10121)

**Supplementary Materials**

**ICD-9 and ICD-10 codes for the mental disorders, COVID-19 outcomes and comorbidities included in the study.**

|  | **Diagnosis** | **ICD-10** | **ICD-9** |
| --- | --- | --- | --- |
| **Mental disorders** | Non-affective psychosis | F20, F21, F22, F23, F24, F25, F28, F29 | 295, 297, 298 (298.0 excluded), 301.22 |
|  | Bipolar disorder | F30, F31 | 296.0, 296.1, 296.4, 296.5, 296.6, 296.7, 296.80, 296.81, 296.89, |
|  | Depressive disorder | F32, F33, F34 | 296.2, 296.3, 298.0, 300.4, 311 |
|  | Stress-related disorders | F43 | 308, 309 |
|  | Neurotic/somatoform disorders | F40, F41, F42, F44, F45, F48, | 300, 300.8 |
|  | Substance misuse | F17 | 305.1 |
|  |  | F10 | 303 |
|  |  | F11, F12, F13, F14, F15, F16, F18, F19 | 304, 305 (305.1 excluded) |
| **COVID-19 outcomes** | COVID-19 hospitalization | Hospitalization caused by U071, B34.2, B97.2, J12.81, J12.89 |  |
| **Comorbidities** | Asthma | J45 |  |
|  | Cardiovascular disease | G45, G46, I63, I65, I66, I67.8, I67.9 |  |
|  | Chronic pulmonary disease | J40, J41, J42, J43, J44, J47 |  |
|  | Diabetes | E10, E11, E12, E13, E14 |  |
|  | Dyslipidemia | E78 |  |
|  | Heart failure | I50 |  |
|  | Hypertension | I10, I11, I12, I13, I15 |  |
|  | Ischemic heart disease | I20, I21, I22, I23, I24, I25 |  |
|  | Neoplasia | All codes beginning by C |  |
|  | Obesity | E66, R63.5, Z68.4 |  |

**Selection of the optimum number of clusters**

K-means is an unsupervised machine learning algorithm used for partitioning a population into K sets or clusters around K centers, making the within-cluster data points as similar as possible while simultaneously keeping the clusters as different (far) as possible. Our purpose here is describing the path we followed to select an optimum number of clusters K (while keeping it reasonably low) into which to partition our data. First, we evaluated the K-means cluster analyses with 2 to 16 clusters. For each clustering, we computed the Calinski-Harabasz index, the Silhouette index (both as an average of cluster silhouettes and also as an average of individual silhouettes), the within-cluster sum of squares , and the logarithm of the sum of squares ratio . The optimal number of clusters is based on the following criteria: the maximum value in the sequence of Calinski-Harabasz indices and in the sequence of Silhouette indices (for 2 to 16 clusters), while for the within-cluster sum of squares and the logarithm of the sum of squares ratio, the maximum difference between consecutive indices (for 3 to 16 clusters).

Then, to determine a prudently number of clusters, we first examined the lowest number of clusters where the sequence of Calinski-Harabasz indices reached a local maximum. We selected this as the optimal number of clusters if all the subsets in the corresponding partition contained at least 30 individuals. This criterion was met with 7 clusters for the group of non-COVID-19 related deaths. If any cluster had fewer than 30 subjects, as occurred with the 16-cluster partition for the group of COVID-19 related deaths, we considered all local maxima of the Calinski-Harabasz and Silhouette indices and the local maxima of consecutive differences for the other selected indices, as shown in the tables. We then tried the related number of clusters using the following procedure:

First, we partitioned for the most common number of clusters in increasing order. For the COVID-19 death cohort these were 12 and 16, both occurring three times (16 clusters did not meet the aforementioned criterion, so the procedure was not retried). Failing to obtain a partition with at least 30 subjects in each cluster, we tried the next most common number of clusters, again in increasing order, which for the COVID-19 death cohort were 6, 9, and 14, each occurring twice. This procedure would have continued until we found a partition satisfying the condition of at least 30 observations per cluster or, failing that, we would have selected the minimum number of clusters that were optimal for some index. However, for the COVID-19 death cohort, the criterion was satisfied with 6 clusters.

Table of local maxima in the sequences of Calinski-Harabaz indices, Silhouette indices, and consecutive differences of within-clusters sum of squares and logarithms of sum of squares ratios for the group of non COVID-19 related deaths.

| Clustering index | Number of clusters | Index value |
| --- | --- | --- |
| Calinski-Harabasz | 7 | 39035.000 |
| Calinski-Harabasz | 15 | 49253.000 |
| Silhouette (as cluster silhouettes average) | 7 | 0.598 |
| Silhouette (as cluster silhouettes average) | 10 | 0.634 |
| Silhouette (as cluster silhouettes average) | 15 | 0.689 |
| Silhouette (as individual silhouettes average) | 10 | 0.690 |
| Silhouette (as individual silhouettes average) | 15 | 0.789 |
| WCSS* | 4 | 4094.000 |
| WCSS* | 7 | 3207.000 |
| WCSS* | 10 | 1688.000 |
| WCSS* | 12 | 410.000 |
| WCSS* | 15 | 1022.580 |
| LogSSRatio* | 4 | 0.203 |
| LogSSRatio* | 7 | 0.174 |
| LogSSRatio* | 10 | 0.114 |
| LogSSRatio* | 12 | 0.029 |
| LogSSRatio* | 15 | 0.116 |

*The index value represents the difference between the index value for the specified number of clusters and the index value for the partition with one cluster less.

Table of local maxima in the sequences of Calinski-Harabaz indices, Silhouette indices, and consecutive differences of within-clusters sum of squares and logarithms of sum of squares ratios for the group of COVID-19 related deaths.

| Clustering index | Number of clusters | Index value |
| --- | --- | --- |
| Calinski-Harabasz | 16 | 310.804 |
| Silhouette (as cluster silhouettes average) | 10 | 0.712 |
| Silhouette (as cluster silhouettes average) | 12 | 0.693 |
| Silhouette (as cluster silhouettes average) | 16 | 0.695 |
| Silhouette (as individual silhouettes average) | 16 | 0.842 |
| WCSS* | 6 | 15.366 |
| WCSS* | 9 | 2.529 |
| WCSS* | 12 | 1.878 |
| WCSS* | 14 | 0.940 |
| LogSSRatio* | 3 | 0.194 |
| LogSSRatio* | 6 | 0.171 |
| LogSSRatio* | 9 | 0.023 |
| LogSSRatio* | 12 | 0.032 |
| LogSSRatio* | 14 | 0.016 |

*The index value represents the difference between the index value for the specified number of clusters and the index value for the partition with one cluster less.

Non COVID-19 death cohort


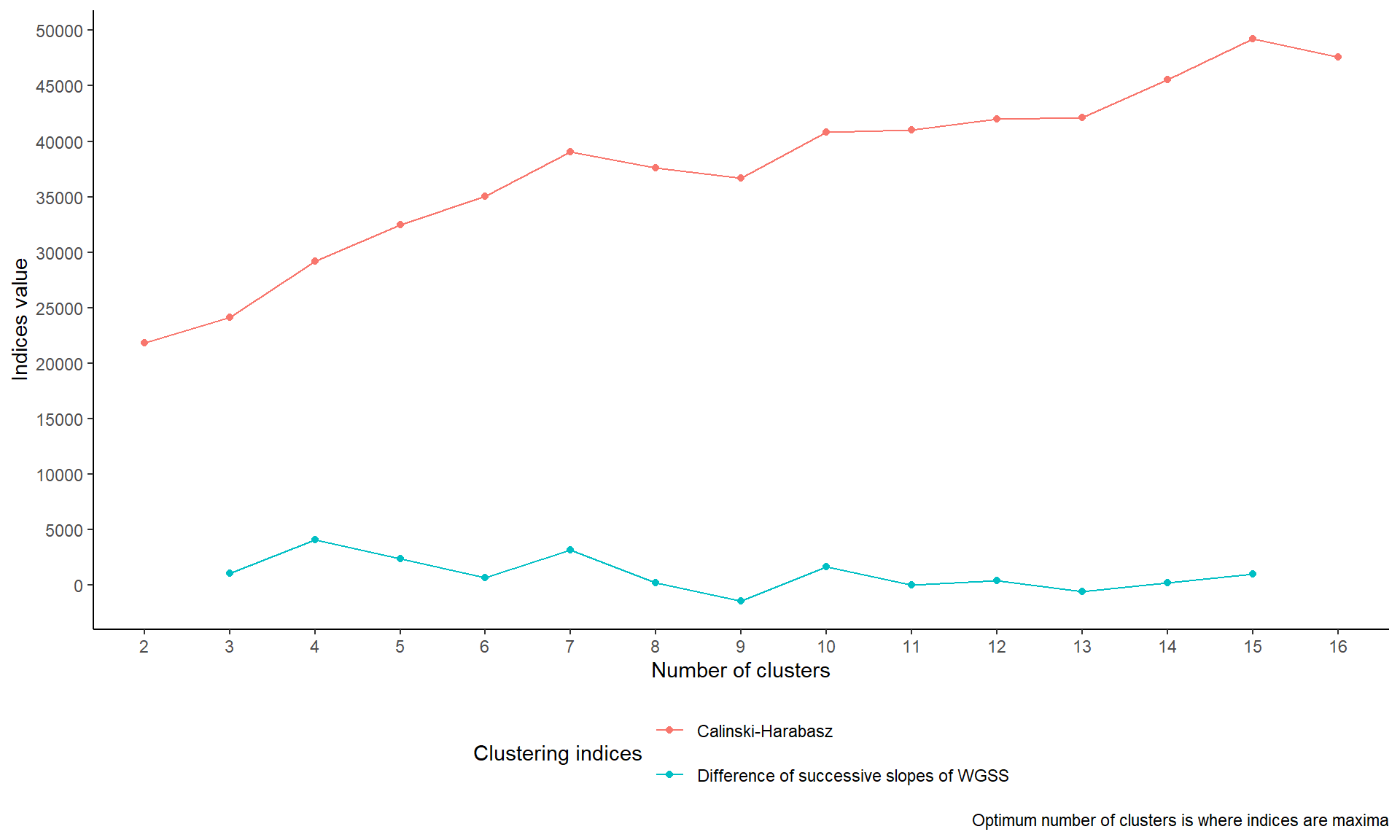


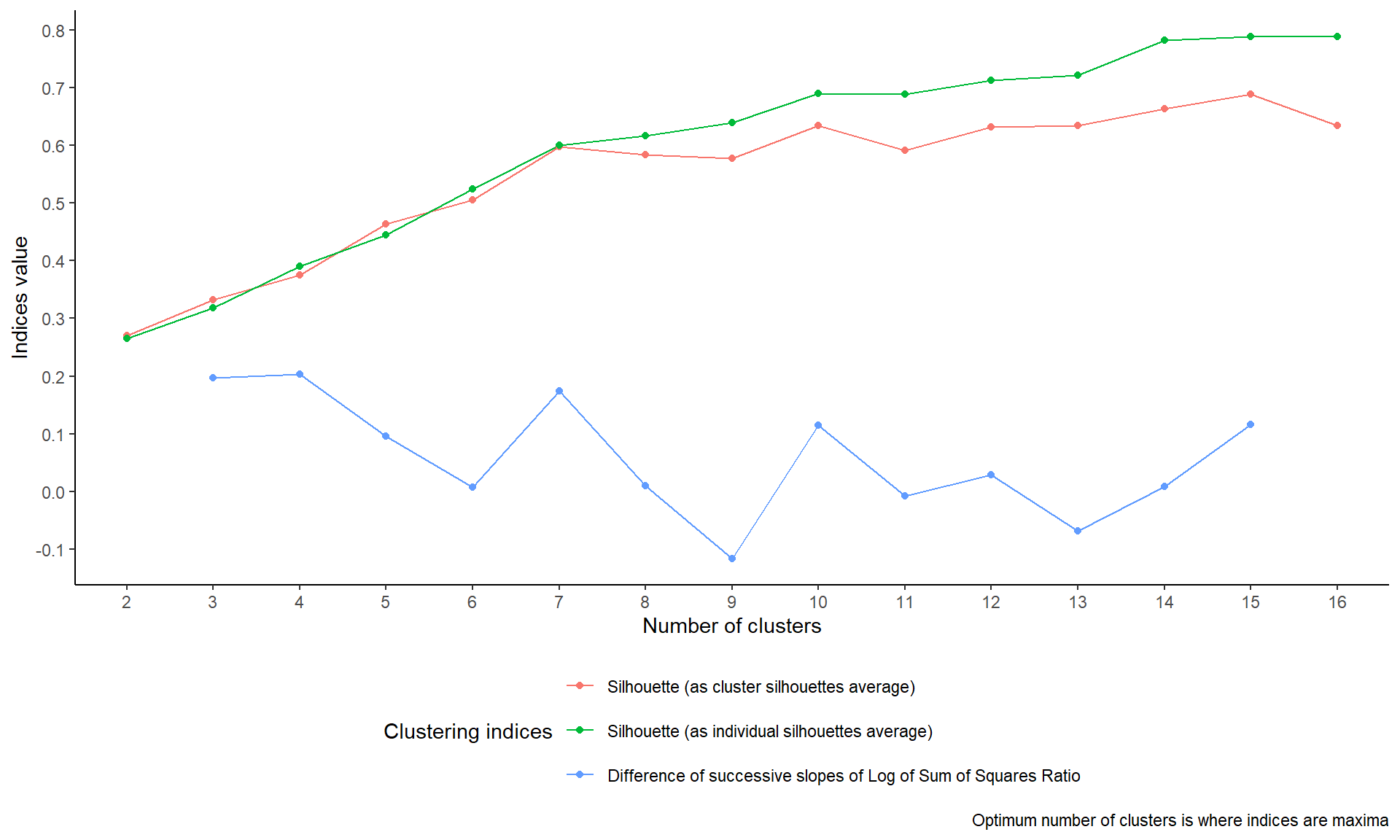


COVID-19 death cohort


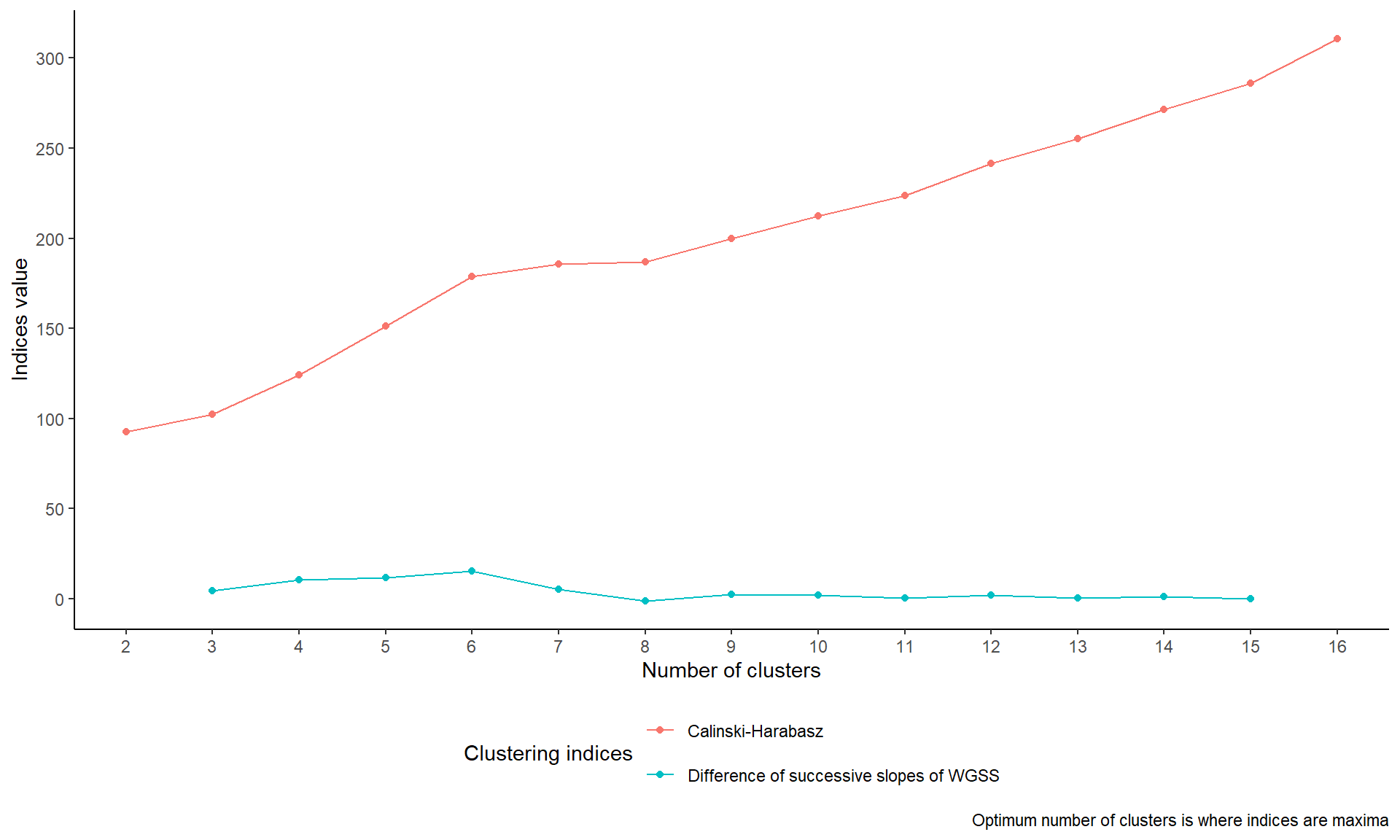


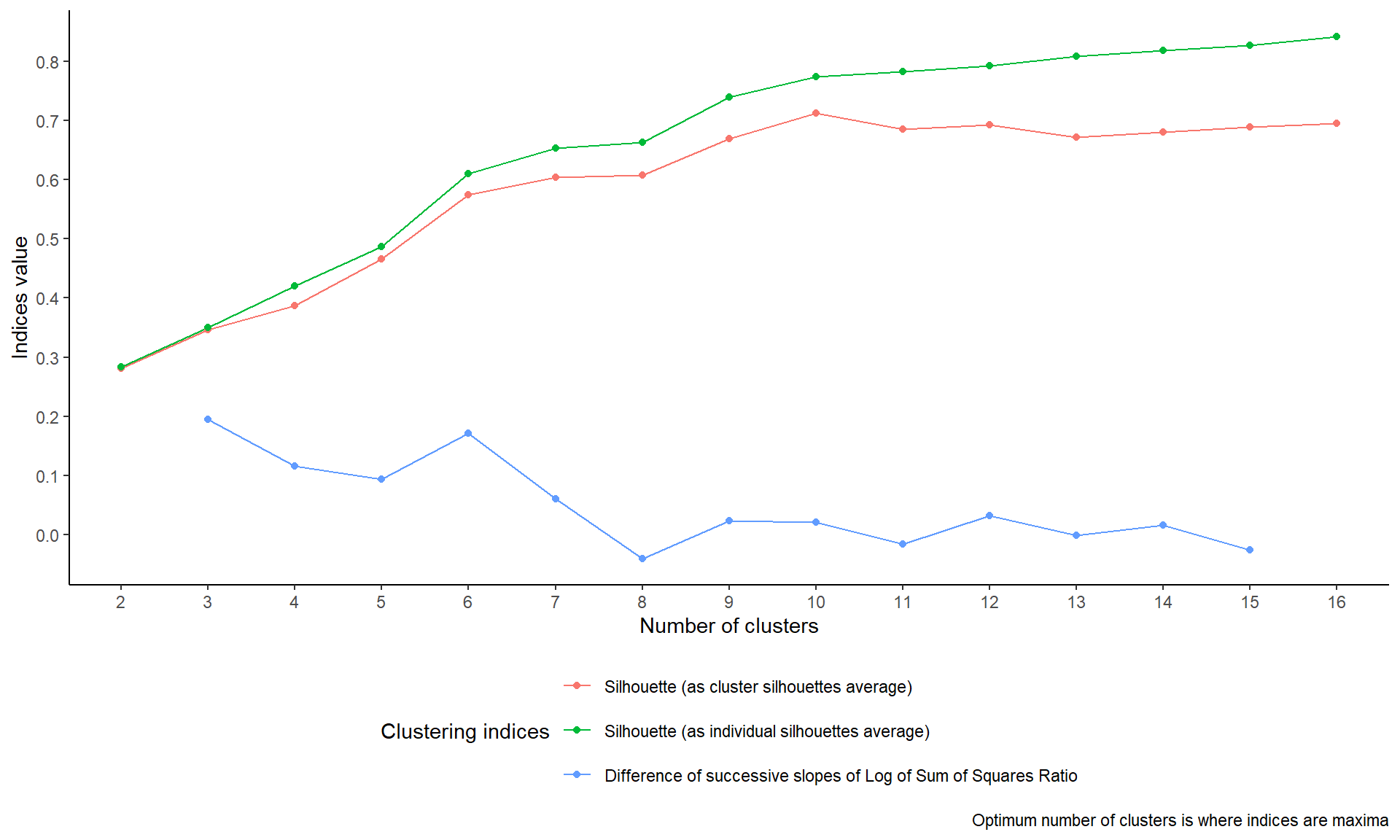


**Supplementary 2**

**Percentage of patients with mental disorders in each cluster**


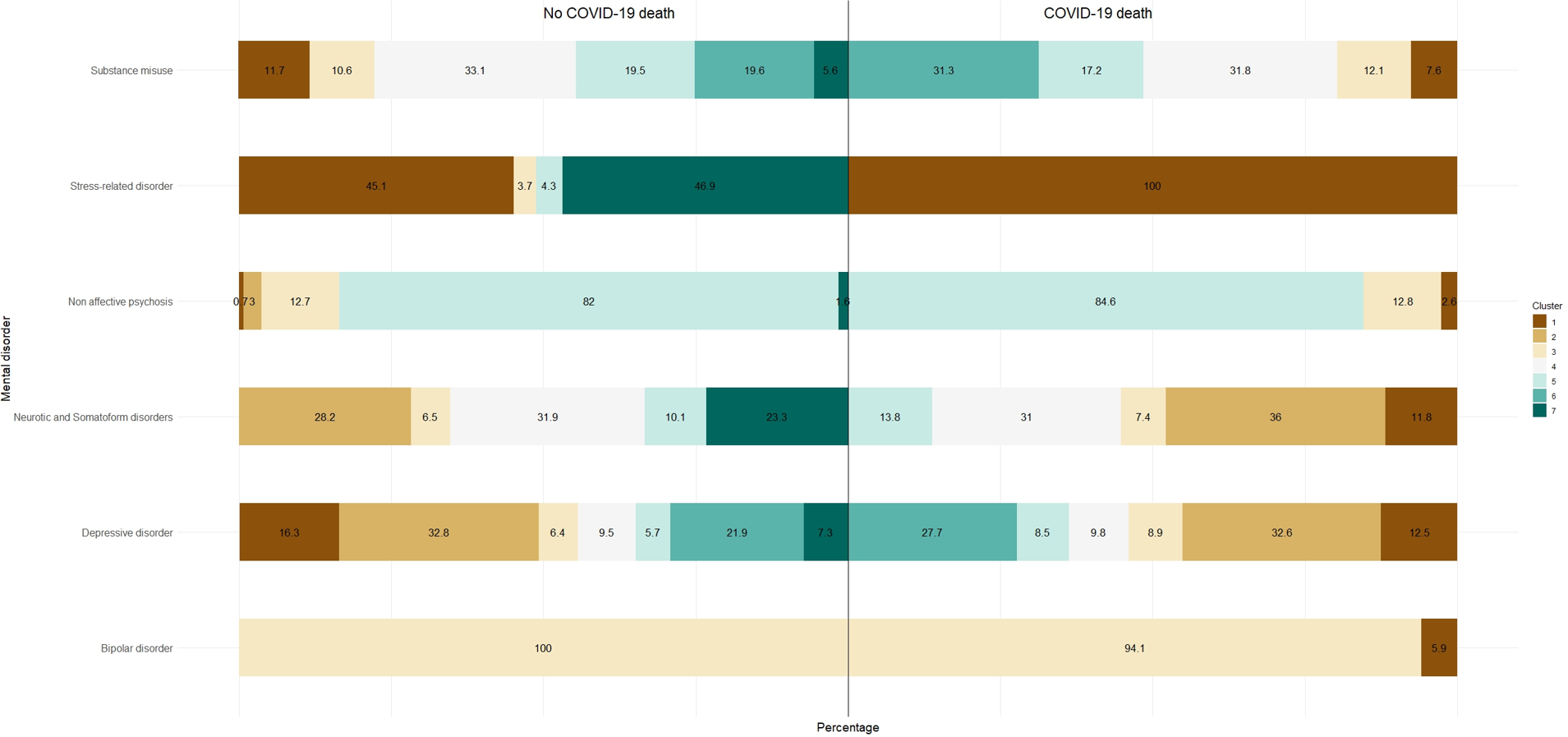

Supplement: Felez-Nobrega et al. supplementary material [file S0924933825101211sup001.docx]
